# Study on the influence of pore dip angle on acoustic emission characteristics of sandstone with composite defects

**DOI:** 10.1371/journal.pone.0337723

**Published:** 2025-11-25

**Authors:** Na Zhao, Yu Wen, Laigui Wang

**Affiliations:** School of Mechanics Engineering, Liaoning Technical University, Fuxin, China; Semnan University, IRAN, ISLAMIC REPUBLIC OF

## Abstract

Rock defects are a key internal factor leading to deformation and failure under load. This study investigates red sandstone specimens with combined pore–fracture defects at different pore dip angles through uniaxial compression tests, while employing Acoustic Emission monitoring to capture the failure process. The evolution of AE characteristic parameters and rock failure modes is analyzed. The results indicate that: (1) the presence of pores prolongs both the time to failure and the onset of the AE burst stage, with longer durations observed at higher pore dip angles; (2) AE signal amplitude and frequency vary significantly across different loading stages, and the b-value exhibits an “increase–fluctuation–decrease” trend, with the decreasing stage serving as a precursor to rock instability; (3) pore dip angle strongly influences crack propagation types: dip angles of 0°–30° favor axial cracks and through-going wing cracks, 45°–75° angles tend to induce co-planar and wing crack connectivity, while 90° angles cause crack deviation, hindering through-going failure; (4) intact rock fails in a tensile–shear mixed mode, whereas the number of shear cracks in rocks with pores initially increases and then decreases with dip angle, reaching a maximum at 45°, resulting in shear-dominated failure. These findings reveal the failure characteristics and AE evolution patterns of rocks with combined pore–fracture defects at different pore dip angles, providing insights for the identification of precursors to rock failure and for disaster prevention and mitigation.

## Introduction

Rock mass, as a complex and diverse natural material, is widely present in various geological engineering and mining activities. Due to the frequent presence of fractures [1], pores [2], and the intrinsic bedding structures of rocks [3], these structural defects not only significantly affect the mechanical properties and stability of fractured rock masses but also cause rocks to exhibit different mechanical behaviors under varying stress conditions [4]. In engineering contexts such as slope projects, tunnel excavation, and underground mining, the uneven spatial distribution of pores and fractures often triggers local or even large-scale overall instability, thereby threatening engineering safety. Therefore, an in-depth study on the influence of combined pore-fracture defects on crack propagation laws in rock masses holds important theoretical significance and engineering reference value.

Both domestic and international scholars have conducted extensive research on rock strength, failure modes, and crack propagation laws. Abbas et al. [5] systematically studied the mechanical behaviors and anisotropic characteristics of weathered shale, sandstone, and their composite samples under different confining pressures and bedding orientations, with a particular focus on revealing the influence of sandstone bedding structures on failure modes. Tian et al. [6] pointed out that, in uniaxial experiments, an increase in fracture inclination led to an exponential increase in compressive strength and elastic modulus; Wang et al. [7] found that both parameters exhibited a “V-shaped” variation trend with inclination angle. Li et al. [8] analyzed the deformation and failure process of fractured rock samples using three-dimensional digital image correlation, and the results indicated that strength, peak strain, and elastic modulus increased with fracture angle. Niu et al. [9] emphasized the significant influence of natural micro-fracture inclination on tensile strength, with failure modes including tensile, shear, and tensile-shear composite failures. Nolen-Hoeksema et al. [10], through prefabricating inclined cracks in marble, discovered that the propagation of new cracks at the crack tip was not symmetric with respect to the prefabricated crack axis. Zhang et al. [11] constructed a defect-containing mechanical model and prepared rock-like specimens, revealing the mechanisms of strength evolution and crack propagation under different defect shapes. Furthermore, Yin et al. [12], Chen et al. [13], Wang et al. [14], and Ma et al. [15] conducted experimental and numerical studies on the effects of pore-fracture combinations on rock failure modes and energy evolution, with results showing that the presence of pores significantly altered crack propagation patterns. Gou et al. [16], based on triaxial tests and numerical simulations of sandstone with symmetrical cracks, also revealed the coupling effect between fracture-pore interaction and failure mode.

Meanwhile, Acoustic Emission technology has become an important tool for studying rock fracture mechanisms. This technology captures transient elastic waves released during crack propagation and plastic deformation [17,18], enabling real-time monitoring and early warning of material damage evolution. Previous studies have shown that AE parameters can effectively characterize rock damage and failure characteristics. Abbas et al. [19] demonstrated that bedding planes and composite structures significantly affect the attenuation of AE energy and crack propagation behavior, while AE can effectively identify key parameters such as crack initiation and crack damage in natural rocks. Wu et al. [20] and Yu et al. [21] revealed, through AE technology, the significant influence of pore and fracture parameters on mechanical properties; Zhang et al. [22] and Dou et al. [23] found that defects such as fractures and pores greatly influence various AE characteristic parameters, particularly AE ring counts, energy, and b-values. Zhang et al. [24] argued that the activity levels of ring counts and cumulative energy can effectively characterize internal specimen damage. In addition, Ou et al. [25,26] further explored the intrinsic relationship between AE time-frequency characteristics and rock mechanical properties, and established a damage model relating AE energy to dissipated energy. The studies of Dong et al. [27] Wang et al. [28] and Zhang et al. [29] also indicated that the decrease in AE b-value is closely associated with imminent material failure and can serve as a precursor to rock instability and failure.

To date, extensive research has been conducted by scholars worldwide on the failure modes and crack propagation mechanisms of rocks containing defects, yielding substantial findings. However, most existing studies have predominantly focused on the effects of fracture orientation, number, geometry, and bedding structure, often treating pores and fractures as a unified entity. This approach tends to overlook the influence of pore spatial position and relative orientation on crack propagation paths and failure modes. Moreover, while current findings generally indicate that mechanical parameters and Acoustic emission characteristics vary linearly with defect properties, it remains unclear whether this linearity holds in the presence of pores. To address this gap, the present study conducts uniaxial compression tests on sandstone specimens containing different pore–fracture combinations. By employing AE monitoring to capture the real-time crack evolution process, this work systematically investigates the mechanical parameters, failure modes, AE characteristics, and source localization patterns under varying pore inclinations, thereby complementing and extending the existing body of research.

## Research method

### Experimental equipment

**Fig 1:**
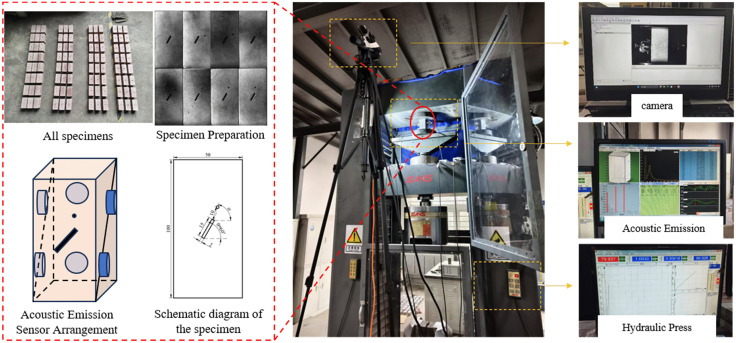
Specimen Preparation and Experimental Setup.

The uniaxial compression experimental setup for rock masses with composite pore-fracture defects consists of three parts: the MTS-600 universal material testing machine loading system and the Acoustic Emission monitoring system. Specific equipment is shown in Fig 1.

The MTS-600 universal material testing machine integrates advanced electromechanical-hydraulic technology. Its main frame uses a high-stiffness gantry structure, equipped with a hydraulic power unit, intelligent control terminal, and data acquisition workstation, enabling fully digital closed-loop control via computer. This system accurately simulates quasi-static compressive loads in the 0 ~ 600 kN range, making it particularly suitable for studying the mechanical responses of brittle materials like geotechnical materials and concrete components. It fully captures key mechanical parameters such as stress-strain curves and elastic modulus. Displacement-controlled loading was applied at a rate of 0.36 mm/min.

The AE signal acquisition system used in this study is a multi-channel AE system from the Laboratory of the Institute of Geology, China Earthquake Administration. The system has 16 channels, and a 6-channel synchronous triggering mode was used in this experiment for full waveform acquisition. Piezoelectric resonant sensors with a frequency response range of 20–400 kHz were employed. Sensor outputs were first amplified by a 40 dB low-noise preamplifier, then digitized by a 6-bit ADC at a sampling rate of 3 MHz, with a sampling length of 4096 points per acquisition. The trigger threshold was set to 40 dB to avoid false triggering from environmental noise. Six sensors were evenly attached to the specimen surface using a dedicated coupling agent to ensure good contact, as shown in Fig 1. After the coupling agent cured, the specimen was placed on the base of the testing machine, and the press head was adjusted to achieve full contact with the specimen surface. Prior to each experiment, calibration was performed at multiple positions on the specimen surface using the standard pencil lead break method to verify inter-channel timing accuracy and relative sensitivity. Calibration results showed that inter-channel timing deviations were less than ±1 µs and amplitude differences did not exceed ±3 dB. After the experiment, the acquired AE data were processed and analyzed, including hit detection, waveform processing, and parameter extraction.

### Specimen preparation

The sandstone used in the experiments was sourced from Jining City, Shandong Province. Following the requirements of the “Standards for Engineering Rock Testing Methods,” original rock blocks were precisely positioned, drilled, and cut using a wire-cutting machine. During specimen preparation, the drilling diameter tolerance was controlled at 3 ± 0.05 mm to ensure the accuracy of pore size. To minimize microstructural damage caused by drilling vibrations, uniform lubrication was applied throughout the drilling process. The spacing of the pores and the relative positions of pores and fractures were strictly arranged according to the design scheme. Standardized rock specimens with dimensions of 50 mm × 50 mm × 100 mm were ultimately prepared.During sample selection, efforts were made to avoid obvious anisotropy and to prioritize homogeneous and dense sandstone to satisfy basic mechanical testing requirements. To highlight the influence of combined pore–fracture defects while minimizing the effect of bedding planes, all specimens were processed to have parallel bedding structures, with the specimen axis perpendicular to the bedding planes. The resulting standardized specimens with combined pore–fracture defects are shown in the left part of Fig 1.

To accurately characterize the mineral composition and microstructural features of the material, X-ray diffraction (XRD) and scanning electron microscopy (SEM) analyses were conducted. The results indicate that the sandstone is primarily composed of albite, plagioclase, calcite, and quartz, with rutile and hematite acting as cementing materials filling the gaps between mineral grains. The prepared rock specimens have a density of approximately 2.4 g/cm³, a porosity of about 12%, an average grain size of approximately 0.25 mm, and a natural moisture content of about 2%

### Experimental equipment

The uniaxial compression tests were conducted in accordance with the Chinese national standard GB/T 23561.4−2009, Methods for Determining the Uniaxial Compressive Strength of Rocks. For the classification of specimens with a single pore, the pore diameter was fixed at 2 mm, the crack width was fixed at 2 mm, and the crack length was fixed at 15 mm. To minimize the influence of the relative position between the pore and the crack on the experiment, the shortest distance between the pore center and the crack was kept constant at 10 mm. The variation of pore position was determined by the angle *α* between the line connecting the pore center to the nearest point on the crack and the horizontal line. The pore variations were conducted on the basis of different crack dip angles, and a two-factor uniaxial compression test was carried out considering both the crack dip angle and the pore–crack angle.

Previous studies have demonstrated that when sandstone contains two combined defects with a relative inclination of approximately 60°, shear–tensile mixed failure is more likely to occur, thereby facilitating crack propagation [30,31]. Building upon this, the present study aims to investigate the influence of pores with different inclinations, located near the potential crack propagation path, on the failure mode of rocks. Accordingly, uniaxial compression tests were conducted on rock specimens with fracture inclinations of 0°, 30°, 60°, and 90°. However, considering space limitations, detailed analysis was focused on specimens with 60° fracture inclinations, as their failure modes and crack propagation paths are more susceptible to pore effects.

### Experimental method

In this study, sandstone specimens containing different pore–crack combination defects were subjected to uniaxial compression tests. The experimental process was monitored using Acoustic Emission technology to investigate the variation laws of AE characteristic parameters under different pore inclination angles. The focus was placed on the AE b-value, the proportion of crack types characterized by RA–AF values, and the correlation between AE source localization and rock fracturing.

(1) Acoustic Emission b-value

In uniaxial compression experiments, the Acoustic Emission b-value is a key parameter used to describe the energy distribution of AE events, and its variation can directly reflect the stages of internal damage evolution in the rock mass as well as the precursory characteristics of failure. At present, the commonly used methods for fitting the b-value are the least squares method and the maximum likelihood estimation method. Although the least squares method is simple and convenient, it has certain limitations—for example, when the number of AE events in the elastic stage is small, it may lead to an artificially high b-value, thus causing distortions due to small sample sizes. In contrast, the maximum likelihood estimation method is more effective, as it can avoid inconsistencies in the weighting of large and small magnitudes and mitigate the problem of insufficient accuracy caused by small samples.

In this study [32], the b-value was estimated using the maximum likelihood method, expressed as:


lgN=a−bM (1)

Where *M* represents the magnitude, which is usually replaced by the Acoustic Emission amplitude(A) divided by 20, i.e., *M* = *A*/20, with units of dB. *N* is the number of AE hits, a is a constant, and b is the AE b-value to be determined. Therefore, the b-value can be calculated using the maximum likelihood estimation method as follows:


$$b={\frac{\mathrm{lg}e}{\bar{M}-M}}_{\min}$$ (2)

Where $\bar{M}$ represents the average magnitude, and $M_{\min}$ denotes the minimum magnitude.

In this study, the total duration of each uniaxial compression test on sandstone specimens with combined pore–fracture defects was divided into 10 time segments [33]. The AE b-value was calculated separately within each segment to obtain the evolution pattern of the b-value throughout a single experiment.

(2) RA–AF values

Du et al. [34] identified that shear failure generates Acoustic Emission events with lower frequencies (typically <150 kHz), whereas tensile failure produces higher-frequency AE events. Consequently, the proportion of distinct frequency bands during rock fracturing can be cross-verified with RA-AF (Rise Angle-Average Frequency) diagrams to determine the ultimate failure mode.

Acoustic emission signals can classify tensile and shear cracks during rock fracturing using two parameters: the average frequency (AF) and the rising angle (RA). This method [35,36] is widely applied in rock mechanics for crack-type identification. The mathematical expressions are defined as follows:


$$RA=\frac{risetime}{amplitude}$$ (3)

$$AF=\frac{counts}{duration}$$ (4)

Here, AF denotes the average frequency of the complete AE waveform, while RA represents the ratio of the rise time (from threshold to peak amplitude) to the peak amplitude.During rock failure, tensile cracks exhibit strong instantaneous characteristics, including short duration, brief rise time, high ring-down counts, and large amplitudes, resulting in low RA values and high AF values. In contrast, shear cracks display opposite traits. According to the JCMS-III B5706 [37] standard, a diagonal line serves as the boundary for distinguishing tensile cracks (above the line) from shear cracks (below the line). By normalizing RA and AF, the crack type can be determined using the AF/RA ratio: tensile cracks when AF/RA > 1, and shear cracks when AF/RA < 1.

(3) Source location

Three-dimensional AE source localization [38,39] can intuitively reflect the initiation location, propagation direction, and spatial morphology of cracks within the rock. During the experiments, AE software was used to monitor crack development in real time. The AE Nano sensors convert the acoustic signals generated by microcrack initiation, propagation, closure, or penetration into electrical signals, which are amplified several times by a signal amplifier before entering the AE system. The amplified signals are then processed to reconstruct the original AE waveforms, which are used to determine the location of damage events.Prior to the experiments, pencil lead break tests were conducted to calibrate the wave velocity, with the measured P-wave velocity of red sandstone approximately 3200 m/s. During localization, the specimen was assumed to be an isotropic medium. Given the relatively dense and homogeneous structure of the rock and the minor influence of bedding planes, a uniform wave velocity was applied. The AIC method was employed for arrival time picking to ensure precise determination of signal start points. Finally, localization accuracy was evaluated by comparing positions obtained from artificial impact points with those calculated using a least-squares inversion method, resulting in an average error of approximately ±2.5 mm.

## Results and analysis

### Stress-strain curve stage division

**Fig 2:**
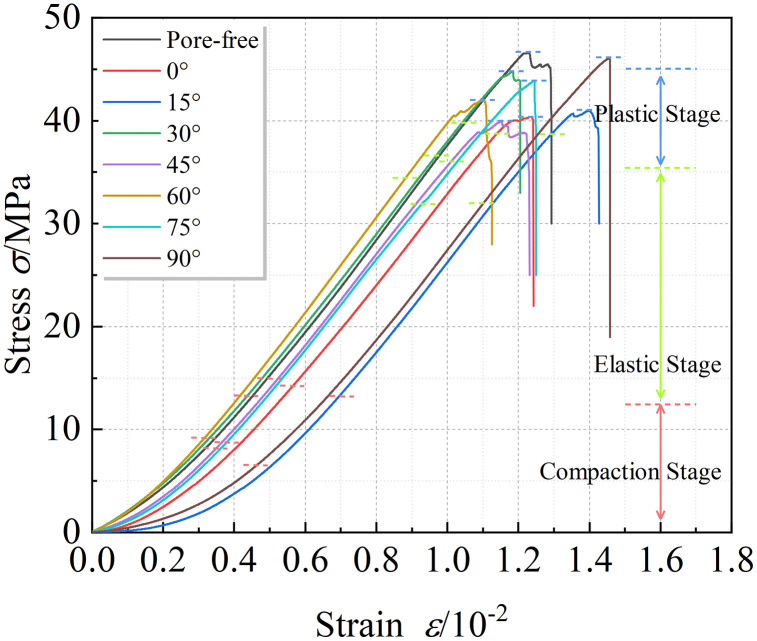
Stress-Strain Curve and Division of Different Stages.

After the uniaxial compression tests on sandstone specimens containing pore–fissure combined defects, the stress–strain curves of each specimen were obtained. Based on the variations in stress and the timing of acoustic emission events, the stress stages were divided accordingly. The specific stress–strain curve is shown in Fig 2.

As shown in Fig 2, the stress-strain curves of rocks with pores at different dip angles can be divided into four stages: compaction stage, elastic stage, plastic stage, and post-peak failure stage. In the compaction stage, the stress-strain curve shows an upward concave trend with gradually increasing slope until stabilization, indicating progressive closure of internal pores and fractures. The elastic stage involves elastic deformation, with crack initiation around pores/fractures due to stress concentration; the curve is linear here, with its slope representing the elastic modulus. In the plastic stage, plastic deformation occurs, cracks propagate and coalesce, and the stress peak is the compressive strength. Finally, in the failure stage, macroscopic cracks penetrate the rock, causing brittle failure with instantaneous stress drop and minimal post-peak phase. The specific mechanical parameters of each specimen are shown in Table 1.

**Table 1:** Mechanical parameters of rocks with combined pore–fissure defects.

Category	Density/(g.cm-3)	E modulus/GPa	Failure strain/%	UCS/MPa
W60	2.457 ± 0.031	4.615 ± 0.149	1.283 ± 0.002	46.733 ± 2.024
K60-0	2.463 ± 0.085	4.432 ± 0.191	1.254 ± 0.022	42.285 ± 3.231
K60-15	2.403 ± 0.021	4.342 ± 0.109	1.277 ± 0.101	40.840 ± 0.899
K60-30	2.487 ± 0.046	4.402 ± 0.121	1.227 ± 0.055	44.878 ± 3.115
K60-45	2.413 ± 0.035	4.387 ± 0.167	1.265 ± 0.160	44.656 ± 4.501
K60-60	2.447 ± 0.076	4.375 ± 0.177	1.197 ± 0.103	42.492 ± 2.934
K60-75	2.410 ± 0.072	4.373 ± 0.270	1.298 ± 0.056	44.016 ± 3.003
K60-90	2.407 ± 0.098	4.419 ± 0.061	1.488 ± 0.024	45.591 ± 3.521

**Fig 3:**
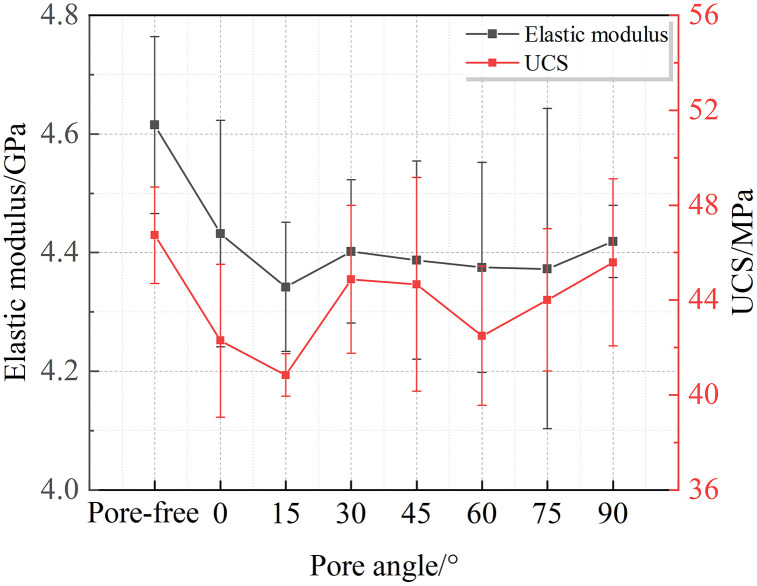
Variation Curves of Compressive Strength and Elastic Modulus.

Fig 3 presents the variation of the average uniaxial compressive strength (UCS) and elastic modulus of rock specimens with combined pore–fracture defects as a function of pore dip angle. It can be observed that the elastic modulus of intact (pore-free) rock remains at a relatively high level. As the pore dip angle increases from 0°, the elastic modulus exhibits an overall decreasing trend, reaching its minimum at a pore dip angle of 15°. Thereafter, the elastic modulus remains relatively stable at a value lower than that of the intact rock, with a slight increase observed when the pore dip angle reaches 90°. This indicates that the presence of pores significantly reduces the elastic modulus of the rock, while variations in pore dip angle have a relatively minor effect.

Meanwhile, the presence of pores also noticeably affects the compressive strength of the rock. For rocks with pores at different dip angles, the UCS exhibits a “V”-shaped variation within the 0°–30° range, first decreasing and then increasing. A similar trend is observed in the 45°–90° range, with the lowest strengths occurring at dip angles of 15° and 60°. This trend aligns well with the variations in time to failure, the AE (acoustic emission) burst stage, and the proportion of shear cracks, indicating that pore dip angle has a comparable influence on different rock failure behaviors.

### AE characteristic evolution analysis

**Fig 4:**
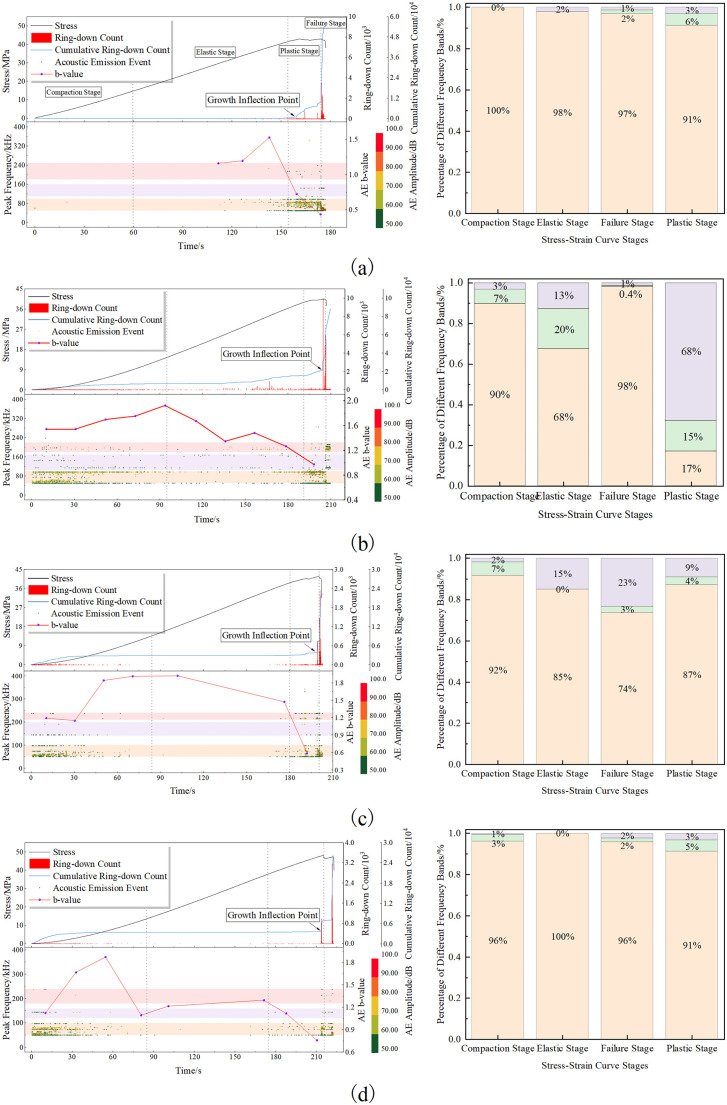
Acoustic emission characteristics and dominant frequency distribution proportions under varying pore inclination angles. (a)Pore-free; (b)*α* = 0°; (c)*α* = 15°; (d)*α* = 30°.

**Fig 5:**
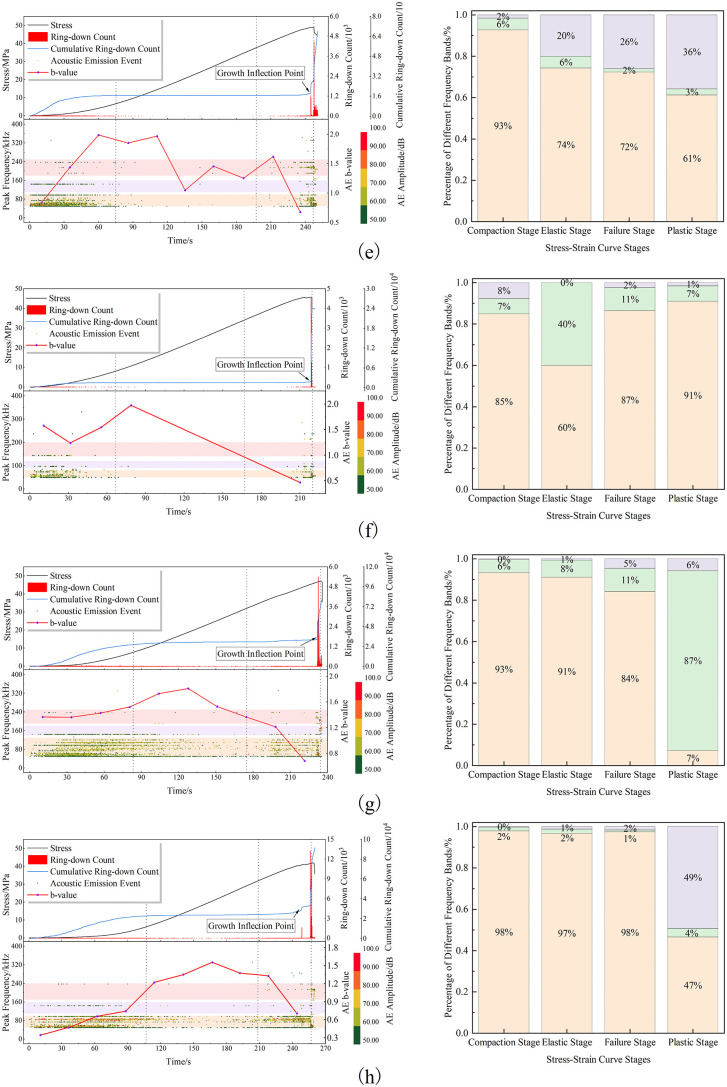
Acoustic emission characteristics and dominant frequency distribution proportions under varying pore inclination angles. (e)*α* = 45°; (f)*α* = 60°; (g)*α* = 75°; (h)*α* = 90°.

Fig 4 and Fig 5shows variations in AE characteristic parameters during uniaxial compression tests under different pore dip angles. The upper left section displays stress-time curves with stage divisions, ring-down counts, and cumulative ring-down counts. The lower left shows AE b-values and distributions of AE events causing b-value changes. The right section shows the proportional distribution of AE events in different frequency bands across stages.

(1) Analysis of Ring-Down Count Variations Across Stages

Based on rock mechanics and stress-strain curves, the stress-time curve is divided into compaction, elastic, plastic deformation, and failure stages. AE ring-down counts can be categorized into three periods: initial period, quiet period, and burst period. The compaction stage corresponds to the initial AE period, dominated by low-amplitude events; cumulative ring-down counts increase nonlinearly then linearly. During the elastic stage, cumulative counts enter a quiet period with few events, remaining nearly flat. Near the end of the elastic stage and start of plastic deformation, AE approaches the burst period, showing stepwise increases. In mid-late plastic deformation, cracks propagate steadily, generating numerous internal cracks; counts enter a burst period with a sharp inflection point near peak strength. AE events surge suddenly, consistent with stress-strain behavior.

(2) Analysis of Rock Fracture Time

From the perspective of individual rock failure time, pore-free specimens exhibited the shortest overall failure duration. In contrast, pore-containing specimens showed prolonged total failure times, with failure duration demonstrating an increasing trend as the pore dip angle increased.The underlying mechanism lies in how pores alter the original failure mode of pore-free specimens, deflecting crack propagation paths. This deviation prevents cracks from following optimal propagation trajectories, and the resultant change in overall failure mode consequently modifies the time required for failure.Based on the rock fracture patterns illustrated in Fig 4 and Fig 5, pore-containing specimens can be broadly categorized into three types:Type I (Pore dip angle *α* = 0° ~ 30°):Failure time increased compared to pore-free specimens.Within this range, failure time exhibited a“V”-shaped trend—initially decreasing then increasing—with rising pore dip angle.Type II (Pore dip angle *α* = 45°~75°):Failure time increasedsignificantlycompared to pore-free specimens.Similarly, failure time showed a”V”-shaped trend(decrease followed by increase) within this range.Type III (Pore dip angle *α* = 90°):Pores exerted an inhibitory effect on crack propagation.Failure duration reached its maximum value.

(3) Analysis of AE Burst Period Timing

The acoustic emission (AE) process of sandstones containing pores with different inclination angles exhibits a generally similar evolution pattern; however, the variation in pore inclination angle also exerts a notable influence on the characteristics of each deformation stage. For the specimen without pores, the AE burst stage occurred at approximately 170 s. When the pore inclination angle *α* ranged from 0° to 30°, the AE burst appeared relatively earlier, occurring around 190–210 s. When *α* was between 45° and 75°, the AE burst occurred later, approximately at 220–240 s. For *α* = 90°, the AE burst appeared the latest, around 250 s. Overall, the occurrence time of the AE burst exhibited a delayed trend with increasing pore inclination angle.

By comparing the AE characteristic curves with the stress–time curves, it can also be observed that the variation trend of the initial stress corresponding to the onset of the plastic deformation stage in rocks with different pore–crack inclination combinations is generally consistent with that of the AE response. Moreover, the occurrence time of the AE burst stage and the time required for rock specimen failure show identical variation trends across different inclination ranges.

(4) Analysis of AE b-value Evolution

Fig 4 and Fig 5 presents the variation curves of Acoustic Emission b-values for rocks with different pore dip angles under uniaxial compression. In subplot (a), b-values could not be calculated during the compaction and initial elastic stages due to insufficient AE event samples. Reliable b-value computation only became feasible in the latter half of the elastic stage when abundant AE events emerged. Similarly, subplot (c) shows missing b-values during the mid-elastic stage owing to limited sample size.Analysis of Fig 4 and Fig 5 reveals that during the compaction stage, AE b-values generally exhibit an upward trend. This phenomenon occurs because external loads compress pre-existing micro-fractures and pores in the rock during early compaction, generating detectable low-amplitude, low-frequency AE signals. As loading progresses to the elastic stage, internal pores and fractures become fully compacted, resulting in structural stabilization. Consequently, AE signals diminish significantly, causing b-values to fluctuate within a narrow range. The sparse AE signals during this stage remain predominantly low-amplitude and low-frequency, similar to those in the compaction stage.Upon entering the plastic stage, extensive micro-cracks initiate and coalesce within the rock, with dominant cracks gradually propagating internally and externally. Continued loading accelerates crack generation, producing abundant medium-to-high amplitude, low-frequency AE signals, while the proportion of medium-to-high frequency signals slightly increases. Correspondingly, b-values undergo accelerated decline, indicating the rock specimen approaches a critical instability state preceding failure. Finally, during the failure stage post peak stress, AE signals are dominated by medium-to-high amplitude and medium-to-high frequency components, heralding sudden macroscopic instability.

Thus, throughout uniaxial compression, AE b-values demonstrate a characteristic “rise-fluctuation-decline” trend: rising during compaction, fluctuating in the elastic stage, and declining through plastic and failure stages. The descending b-value trajectory in the plastic stage serves as a key precursor for rock instability. However, limitations arise from its time-windowed statistical averaging nature: while improper time-window selection may cause significant statistical fluctuations, the overall trend of b-values remains an effective and direct indicator of rock fracture evolution.

### Analysis of RA-AF value variations

**Fig 6:**
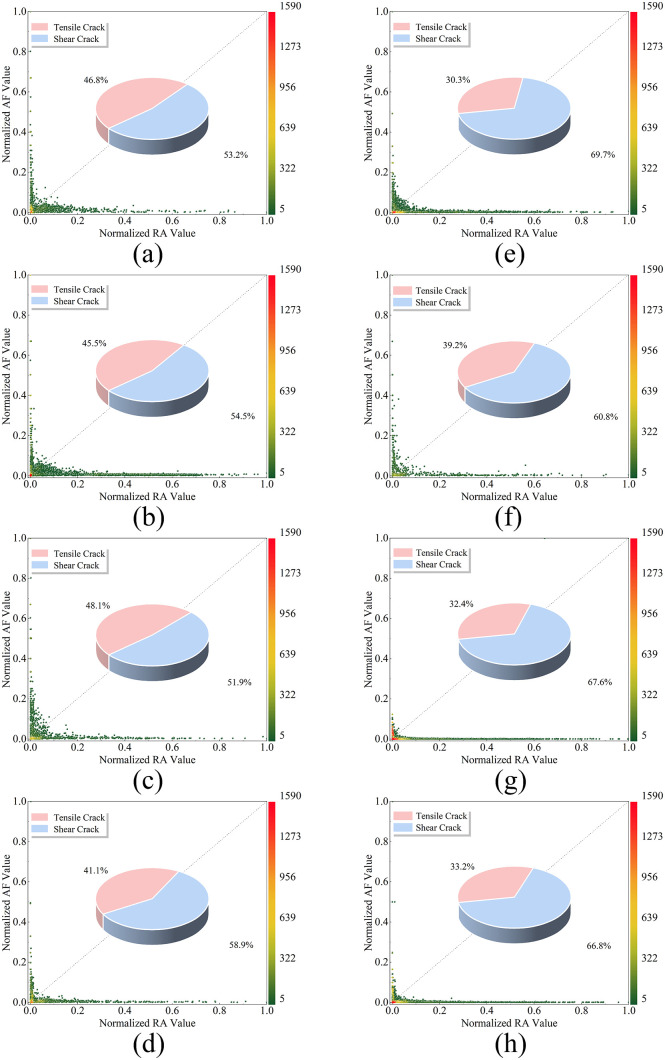
Normalized RA-AF value distribution under varying pore inclination angles. (a)Pore-free; (b)*α* = 0°; (c)*α* = 15°; (d)*α* = 30°; (e)*α* = 45°; (f)*α* = 60°; (g)*α* = 75°; (h)*α* = 90°.

Fig 6 illustrates the normalized RA-AF distributions for sandstone with varying pore inclination angles.

Fig 6 displays normalized RA-AF value distributions under varying pore dip angles. Analysis reveals that for rock specimens containing 60°-inclined fissures,the dominant failure mode transitions from tensile-shear hybrid to predominantly inclined shear failure as pore dip angle increases. Regardless of pore presence, shear cracks consistently outnumber tensile cracks during testing, with the shear-to-tensile ratio initially increasing then decreasing as pore dip angles enlarge.

For 60°-inclined fissures in pore-free specimens, shear cracks slightly exceed tensile cracks due to the characteristic “X”-shaped conjugate shear failure typical of sandstone under uniaxial compression. Across all configurations, tensile crack counts exhibit a “decrease-increase” transition pattern. Crucially, as pores approach the initial crack propagation path with increasing dip angles, shear cracks progressively multiply, peaking at 45° pore inclination before declining, while tensile cracks conversely increase.At 90° pore inclination, although pores and fissures remain non-intersecting, they vertically extend partial tensile cracks, reducing the overall shear crack ratio without altering the ultimate failure mode.

Fundamentally, pores redirect crack propagation toward stress-concentrated zones surrounding them, consistently biasing crack paths toward pore locations. This steering effect intensifies as the spatial relationship between pores and fissure tips (crack initiation points) evolves from relatively parallel to perpendicular orientations, thereby dictating the observed failure mode transitions.

### AE specimen failure modes

**Fig 7:**
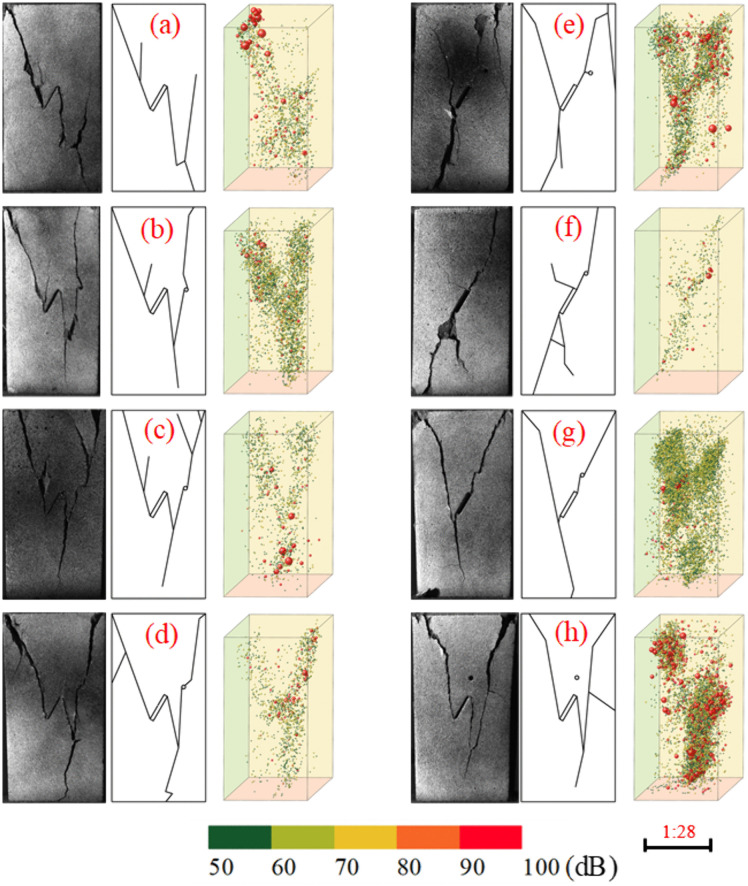
Specimen fracture and acoustic emission source location map. (a)Pore-free; (b)*α* = 0°; (c)*α* = 15°; (d)*α* = 30°; (e)*α* = 45°; (f)*α* = 60°; (g)*α* = 75°; (h)*α* = 90°.

Fig 7 presents schematic diagrams of rock specimen fracture patterns, crack propagation sketches, and acoustic emission source localization distributions. The color gradient transitioning from green to red represents increasing magnitudes of acoustic emission events, while the dot sizes proportionally scale with the magnitude values.

(1) Rock Failure Mode Analysis

Fracture morphologies in porous rock specimens are classified into two distinct patterns based on pore dip angles (*α*): Type I for *α* = 0° ~ 30° and Type II for *α* = 45° ~ 75°. At *α* = 90°, pores alter propagation paths of flaw-initiated cracks but prevent direct coalescence between pores and pre-existing fractures.

In pore-free specimens, anti-wing cracks initiate from the upper and lower tips of pre-existing flaws, propagating obliquely toward the specimen boundaries. A secondary vertical crack subsequently nucleates at the midspan of the lower anti-wing crack, ultimately leading to through-going failure. For specimens with pore dip angles *α* = 0° ~ 30° (Type I morphology), the final fracture pattern resembles that of pore-free specimens: cracks initiate radially from flaw tips and pore peripheries, with flaw-tip cracks evolving as anti-wing cracks. Tensile cracks propagate radially along the loading direction above pores, while secondary cracks below pores extend obliquely to penetrate the anti-wing cracks, forming conical failure blocks. This behavior is attributed to pore guidance effects: increasing *α* induces counterclockwise deflection of secondary cracks penetrating anti-wing cracks. At *α* = 30°, these deflected cracks approach vertical tensile fractures, causing near-axial spalling failure on the specimen’s right side where pores are located.

Specimens exhibit similar fracture patterns distinct from pore-free cases, characterized by wing-crack-dominated oblique penetration. Wing cracks initiating from upper flaw tips propagate toward pores, penetrating them before nucleating coplanar secondary cracks. Ultimately, upper and lower cracks coalesce to form triangular fragments. Within *α* = 45° ~ 75°, proximity of pores to wing-crack paths accelerates their propagation and suppresses anti-wing crack development on the specimen’s right side. This causes near-coincidence of secondary cracks with wing cracks, fundamentally altering failure modes. At *α* = 60°, pore-induced acceleration peaks: wing cracks merge with pores along their paths while anti-wing crack propagation nearly ceases, forming a continuous oblique main crack through coalescence with coplanar secondary cracks, splitting specimens into two triangular fragments. At *α* = 90°, crack propagation resembles Type I morphology but lacks axial tensile cracks above pores due to absence of pore obstruction. Consequently, failure transitions from tensile-shear hybrid to shear-dominated mode. As seen in Fig 7(h), although pores remain uncracked, crack paths deflect significantly toward them, confirming persistent pore guidance effects that disrupt normal crack coalescence. This demonstrates pore-induced variations in fracture morphology and crack propagation laws within rock specimens.

(2) AE Source Location Analysis

Three-dimensional Acoustic Emission source localization (Wang et al. 2021; Zhao et al. 2025) provides direct visualization of crack nucleation sites, propagation trajectories, and spatial configurations within rock specimens. In intact specimens, microcracks predominantly nucleate near specimen ends and propagate inward along the loading direction. Conversely, in pre-cracked specimens, microcracks initiate from the tips of pre-existing flaws and propagate radially outward at characteristic angles.

Three-dimensional acoustic emission source localization analysis of pore-flaw specimens under uniaxial compression reveals a predominance of low-to-moderate amplitude events with scarce high-amplitude signals. During the compaction stage, widespread closure of internal microcracks generates abundant low-amplitude events distributed throughout the specimen alongside limited high-amplitude events. The elastic stage exhibits fewer acoustic emission events, predominantly of low-to-moderate amplitude concentrated at stress concentration zones surrounding pores/flaws and flaw tips where microcracks initiate. Upon entering the yielding stage, coalescing microcracks trigger a surge of moderate-to-high amplitude events clustered along developing primary crack paths. Post-peak failure demonstrates explosive growth of high-amplitude events, though their spatial distributions vary significantly across specimen types: in pore-free and Type I fracture morphology specimens (*α* = 0° ~ 30°), high-amplitude events localize at tips of vertically penetrating main cracks, indicating flaw-dominated failure with minimal pore influence; whereas in Type II specimens (*α* = 45° ~ 75°), these events concentrate around pores and midspans of main cracks, reflecting pore-enhanced failure mechanisms where crack coalescence depends critically on pore-flaw interactions, demonstrating substantially intensified pore guidance effects compared to Type I. At *α* = 90°, pores deflect crack paths without promoting coalescence, ultimately disrupting failure patterns—unlike pore-free specimens, cracks exhibit limited propagation before catastrophic rupture occurs abruptly at peak stress, with high-amplitude events proliferating extensively around both cracks and pore-flaw systems during failure.

Consequently, the fracture processes in rock specimens exhibit distinct variations depending on pore inclination angles, where the pore guidance effect intensifies progressively with increasing *α*, reaching its maximum efficacy within the 60° ~ 75° range. At *α* = 90°, however, this effect reverses—pores disrupt fracture propagation through deflection mechanisms rather than facilitating crack coalescence.

## Discussion

Internal damage evolution constitutes a fundamental mechanism governing rock fracture. The time-to-failure parameter quantifies the temporal scale of transition from steady-state damage accumulation to unstable rupture, simultaneously characterizing pore-induced variations in crack propagation velocity. The onset of Acoustic Emission burst periods marks critical thresholds of accelerated internal damage, where their temporal delay reflects the inhibitory effect of pores on damage accumulation rates—manifested through the progression from decentralized microcrack nucleation to concentrated coalescence. Throughout fracture processes, internal damage evolves via crack propagation and coalescence, with shear/tensile crack ratios revealing pore inclination-dependent failure modes. These modes inherently correspond to distinct crack propagation velocities and internal damage rates.

**Fig 8:**
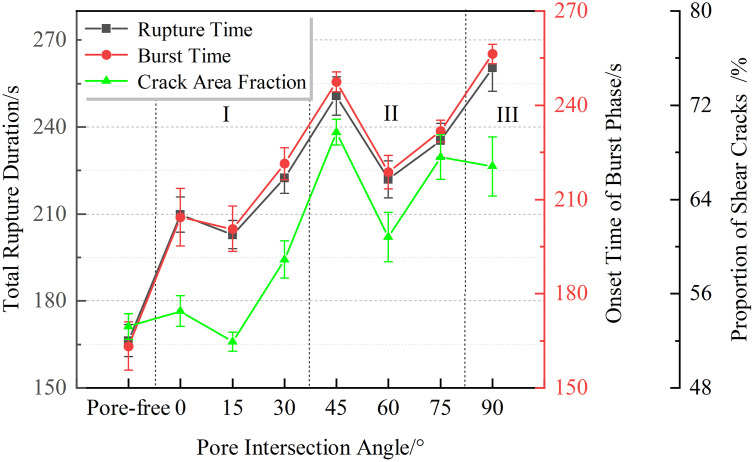
Similarity of Effects Induced by Different Pore Inclination Angles.

Consequently, divergent trends emerge across key parameters—including time-to-failure, AE burst initiation timing, and shear crack evolution—demonstrating angle-specific pore influences as quantitatively illustrated in Fig 8.

Based on Fig 8 depicting fracture patterns in rock specimens with varying pore inclination angles, three distinct failure modes are classified. This categorization reveals nonlinear influences of pores on acoustic emission characteristics and crack-type ratios across different angular intervals.

### “V”-shaped trend in interval I (*α*=0°~30°)

**Fig 9:**
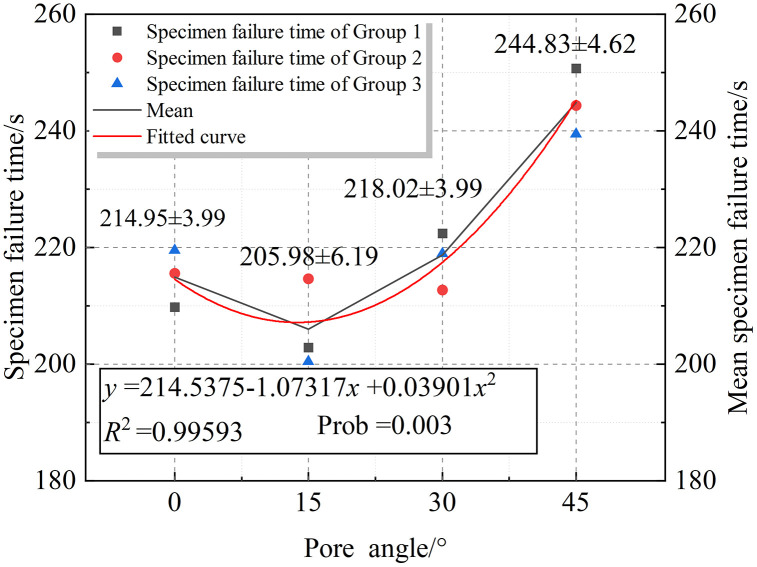
Fitted curve of specimen failure time within the Type I interval.

In the range of pore angles between 0° and 45°, the rock fracture time exhibits a “V-shaped” variation, decreasing first and then increasing. The specific fitted curve is shown in Fig 9.

Fig 9 shows the fitting curve and its quantification of the rock failure time within the Type I interval. When the pore inclination angle is in the range of 0–45°, the overall trend exhibits a “V-shaped” variation. A quadratic polynomial fitting was applied, and the resulting fitting curve is given as:


$$y=214.5375-1.07317x+0.03901x^{2}$$ (5)

The coefficient of determination is *R*² = 0.99593, indicating that the overall fitting effect is highly reliable. Moreover, the p-value is 0.003, suggesting that the fitting is statistically significant. This demonstrates that, within the range of 0°–45°, there exists a significant quadratic relationship between rock failure time and pore inclination angle.

Under pore-containing specimen conditions, rock primarily fractures in a mixed shear-tensile mode. Since tensile cracks align parallel to the principal stress direction and dominate the fracture pattern, energy dissipation during rock failure is minimized. Consequently, both the time-to-failure and the onset of Acoustic Emission burst periods occur earliest.

When the pore inclination angle approaches 0°, pores and fissures become nearly parallel. Cracks initiate from fissure tips and pore ends, subsequently propagating axially to form secondary cracks. These near-vertical propagation paths result in higher energy dissipation compared to pore-free specimens, extending damage accumulation time. Thus, pore presence fundamentally alters the overall fracture mode.At a pore inclination of ~15°, the orientation connecting pores and fissures deviates from the principal stress direction. This angular mismatch deflects crack paths toward greater inclination angles, resembling counter-wing cracks originating from fissures. The shortened propagation paths enhance efficiency, reducing time-to-failure.When pore inclination reaches ~30°, crack deflection angles further increase. Extended propagation paths elevate energy dissipation, causing time-to-failure to rebound. These mechanisms collectively produce a “V”-shaped trend in all parameters across the *α* = 0° ~ 30° range.

### “V”-shaped trend in interval II (*α*=45°~75°)

**Fig 10:**
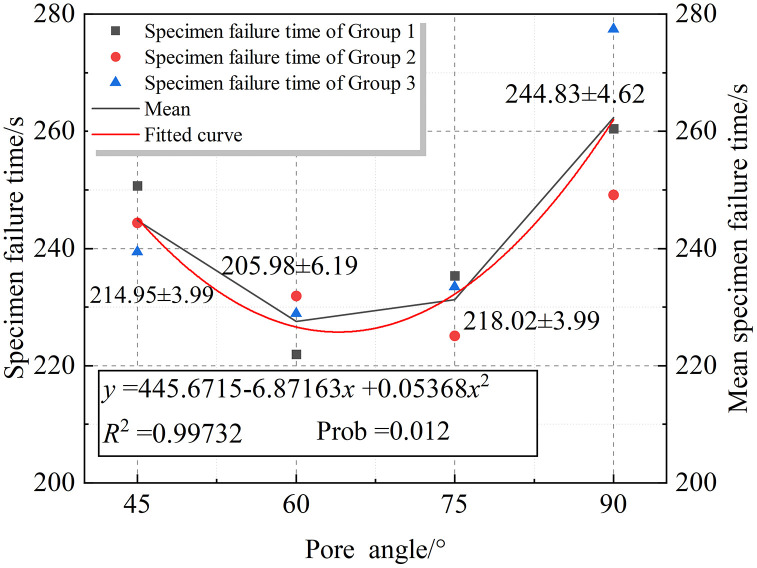
Fitted curve of specimen failure time within the Type II interval.

In the range of pore angles between 45° and 90°, the rock fracture time also shows a “V-shaped” variation pattern, decreasing first and then increasing, rather than a linear trend. The specific fitted curve is shown in Fig 10.

Fig 10 presents the fitting curve and its quantification of rock failure time within the Type II interval. In the range of 45°–90° pore inclination, the overall trend exhibits a “V”-shaped variation. A quadratic polynomial fitting was performed, and the resulting fitting curve is expressed as:


$$y=445.6715-6.87163x+0.05368x^{2}$$ (6)

The coefficient of determination is *R*² = 0.99732, indicating that the overall fitting is highly reliable. The p-value is 0.012, showing that the fitting is statistically significant. This indicates that, within the range of 45°–90°, there exists a distinct nonlinear relationship between rock failure time and pore inclination angle.

As pore inclination angles further increase, the fracture morphology of rock undergoes fundamental changes. The dominant failure mode shifts from counter-wing crack penetration in pore-free specimens to oblique penetration by wing cracks. Consequently, fracture time exhibits a slight increase within this angular range.

Under these conditions, pore positions lie within the strategic zone for crack propagation. At inclinations of 45° ~ 60°, pronounced shear stress concentration occurs at pore peripheries. Crack paths align precisely with shear stress directions, propagating rapidly along maximum shear stress trajectories. This reduces energy dissipation during failure, leading to a gradual decrease in fracture time with increasing inclination.When inclination reaches 75°, pores approach vertical alignment. Crack paths become more perpendicular, with tensile cracks significantly increasing. Cracks initiate above and to the left of pores, where shear and principal stresses concentrate around pore boundaries. These paths deviate inversely from the optimal fracture trajectory while extending in length, intensifying energy dissipation. Consequently, fracture time rebounds.Collectively, these mechanisms produce a V-shaped trend in all parameters across the *α* = 45° ~ 75° range.

### Effect in interval III (*α*=90°)

At *α* = 90°, pores are perpendicularly positioned above fissures. Crack propagation completely bypasses the pores, while symmetrically opposing effects emerge on both sides of each pore. This configuration exerts an inhibitory effect causing reverse deflection on crack extension from both lateral directions. Energy dissipation intensifies through extensive microcrack propagation, resulting in peak accumulated dissipated energy. Consequently, fracture time reaches its maximum.

Across all angular intervals, the observed V-shaped trends consistently exhibit the following pattern: the trough corresponds to the optimal crack propagation angle within each interval; the rising limb reflects increased crack path deviations and elevated energy dissipation induced by pore interference; while the peak signifies maximal inhibitory effects of pores on crack advancement.

### Comparison with existing studies

**Fig 11:**
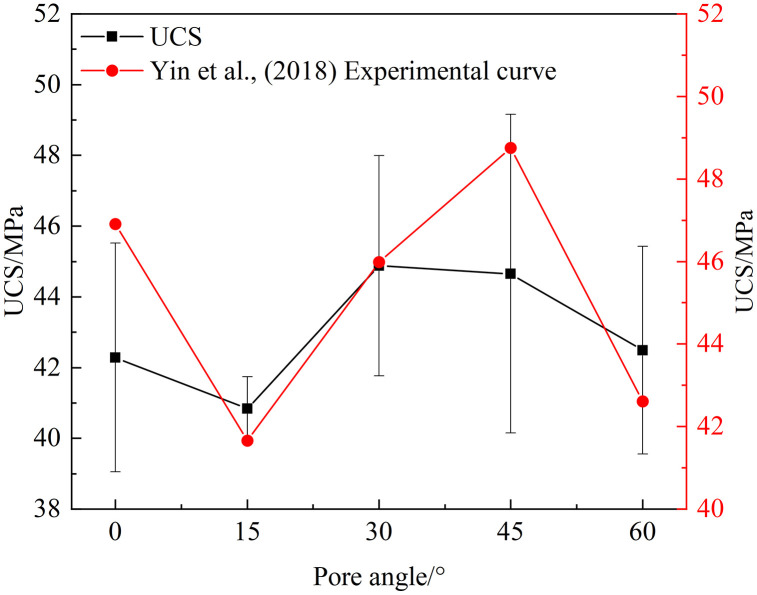
Comparison with Existing Studies.

Numerous studies have investigated the mechanical behavior and Acoustic Emission characteristics of rocks containing defects, generally concluding that combined pore–fracture defects significantly influence rock strength, failure modes, and AE evolution [4], with decreases in b-values often regarded as important precursors to rock instability [40]. These conclusions are broadly consistent with the overall trends observed in this study. However, most existing studies treat pores and fractures as a single entity or examine only a single type of defect [41], paying limited attention to the effect of pore spatial location and its relative orientation with fractures on crack propagation paths and failure modes. Yin et al. [42] found that under the same combined pore–fracture defect conditions, using the pore as a base defect and rotating the fracture as a variable, the variation in inclination between the pore and fracture led to minimum rock strength at 15° and 60°, exhibiting a “V-shaped” trend. However, in that study, the crack propagation path and overall failure mode were mainly controlled by the fracture, making it difficult to reflect the intrinsic role of the pore itself. The specific comparison is shown in Fig 11.In this study, fracture-containing rock specimens were used as the base, and pore dip angle was treated as the experimental variable to focus on the guiding effect of pores on crack propagation. The results indicate that pores not only alter the crack initiation path but also induce pronounced nonlinear variations in AE characteristic parameters, crack proportion, and failure time. These findings not only supplement and extend previous research but also further highlight the unique role of combined pore–fracture defects in understanding rock failure mechanisms.

## Conclusions

This study investigated the influence of pore inclination on crack propagation and acoustic emission (AE) characteristics in sandstone specimens containing pore–fissure combined defects through uniaxial compression tests. The main conclusions are as follows:

(1) The presence of pores delays the rock fracture process by guiding crack deflection and extending the duration of damage accumulation.
(2) AE parameters exhibit stage-dependent variations, and the overall trend of the b-value follows a pattern of “increase–fluctuation–decrease,” effectively characterizing the compaction, elastic, plastic, and failure stages of the rock. The decline in b-value can serve as a precursor indicating imminent instability and failure.
(3) The pore inclination angle significantly affects crack propagation paths and fracture modes: low inclination angles promote the development of secondary cracks, medium to high angles guide the deviation of wing cracks, while vertical pores alter the crack direction but are less likely to cause through-going fractures.
(4) Pore inclination regulates fracture efficiency by influencing crack initiation and energy dissipation. Low to moderate inclination angles correspond to efficient stages of rapid crack propagation, whereas vertical pores result in increased energy dissipation and delayed failure.

## Supporting information

S1 Data UCS texts data. (XLSX)
